# Nature vs. Nurture: Defining the Effects of Mesenchymal Stromal Cell Isolation and Culture Conditions on Resiliency to Palmitate Challenge

**DOI:** 10.3389/fimmu.2019.01080

**Published:** 2019-05-10

**Authors:** Lauren K. Boland, Anthony J. Burand, Devlin T. Boyt, Hannah Dobroski, Lin Di, Jesse N. Liszewski, Michael V. Schrodt, Maria K. Frazer, Donna A. Santillan, James A. Ankrum

**Affiliations:** ^1^University of Iowa Fraternal Order of Eagles Diabetes Research Center, University of Iowa, Iowa City, IA, United States; ^2^Roy J. Carver Department of Biomedical Engineering, University of Iowa, Iowa City, IA, United States; ^3^Department of Obstetrics and Gynecology, Center for Immunology and Immune Based Diseases, Center for Hypertension Research, University of Iowa, Iowa City, IA, United States

**Keywords:** mesenchymal stromal cells, umbilical cord mesenchymal stromal cells, obesity, palmitate, human platelet lysate, fetal bovine serum, culture media, cell therapy

## Abstract

As MSC products move from early development to clinical translation, culture conditions shift from xeno- to xeno-free systems. However, the impact of isolation and culture-expansion methods on the long-term resiliency of MSCs within challenging transplant environments is not fully understood. Recent work in our lab has shown that palmitate, a saturated fatty acid elevated in the serum of patients with obesity, causes MSCs to convert from an immunosuppressive to an immunostimulatory state at moderate to high physiological levels. This demonstrated that metabolically-diseased environments, like obesity, alter the immunomodulatory efficacy of healthy donor MSCs. In addition, it highlighted the need to test MSC efficacy not only in ideal conditions, but within challenging metabolic environments. To determine how the choice of xeno- vs. xeno-free media during isolation and expansion would affect future immunosuppressive function, umbilical cord explants from seven donors were subdivided and cultured within xeno- (fetal bovine serum, FBS) or xeno-free (human platelet lysate, PLT) medias, creating 14 distinct MSC preparations. After isolation and primary expansion, umbilical cord MSCs (ucMSC) were evaluated according to the ISCT minimal criteria for MSCs. Following baseline characterization, ucMSC were exposed to physiological doses of palmitate and analyzed for metabolic health, apoptotic induction, and immunomodulatory potency in co-cultures with stimulated human peripheral blood mononuclear cells. The paired experimental design (each ucMSC donor grown in two distinct culture environments) allowed us to delineate the contribution of inherent (nature) vs. environmentally-driven (nurture) donor characteristics to the phenotypic response of ucMSC during palmitate exposure. Culturing MSCs in PLT-media led to more consistent growth characteristics during the isolation and expansion for all donors, resulting in faster doubling times and higher cell yields compared to FBS. Upon palmitate challenge, PLT-ucMSCs showed a higher susceptibility to palmitate-induced metabolic disturbance, but less susceptibility to palmitate-induced apoptosis. Most striking however, was that the PLT-ucMSCs resisted the conversion to an immunostimulatory phenotype better than their FBS counterparts. Interestingly, examining MSC suppression of PBMC proliferation at physiologic doses of palmitate magnified the differences between donors, highlighting the utility of evaluating MSC products in stress-based assays that reflect the challenges MSCs may encounter post-transplantation.

## Introduction

Cell bioprocessing, involving the large scale production of high volumes of consistent and efficacious cellular products, is a critical component for the translation of mesenchymal stromal cell (MSC) products from academic laboratories to clinical application (1–4). However, a mismatch in culture conditions between basic academic research and clinical translation often exists, with academic labs often relying on fetal bovine serum (FBS) supplemented media and small 2D culture systems, while clinical-grade products are grown in large batches in xeno-free medias (1, 2, 5, 6). Decisions that may once have seemed trivial or even arbitrary in the academic research environment [e.g., media composition (7–11), cell culture format (2, 12–14), or substrate-stiffness (15, 16)] become critical parameters to control in the large-scale industrial production of clinical-grade cellular therapies (17–19). Increasingly, as cellular therapies have been used to treat patients in clinical trials, it has become clear that adopting good manufacturing practices (GMP) early at the research phase can hasten the transition from the bench to the clinic (2, 4, 19, 20). With the diversification of the MSC field to include MSCs harvested from a number of tissue sources (21–24), it is critical to distinguish variance that is inherent in the cell source vs. variability that is introduced by differences in bioprocessing during initial isolation and *in vitro* expansion (25).

As findings from the lab transition to the clinic, a number of process-related changes are often needed to produce a clinical-grade product (2, 3, 18). One of the most glaring differences between MSCs studied in research labs to those produced at an industrial scale for use in patients is a switch from using animal serum as a growth supplement to xeno-free alternatives like human platelet lysate or chemically defined serum (6, 26). There is significant and valid concern surrounding the use of animal derived products in the generation of clinical-grade cell therapies (6, 27, 28). Consequently, transitioning to clinical-grade production often involves transitioning to a xeno-free culture system (1, 2). Today, a large number of xeno-free alternatives are available, many of which have associated drug master files submitted to the FDA, which can make submission of investigational new drug (IND) applications more streamlined (6, 26, 29). A number of notable advantages have been reported for xeno-free culture systems for MSCs, including enhanced cell yield (9, 10, 30), rapid growth kinetics (5, 10), elimination of xenogeneic pathogens (27), and improved genetic stability over extended culture periods (30, 31). In order to ensure that the transition from pre-clinical to clinical application is successful, it is important to understand how the process differences related to culture environments affect MSC function.

Variability in MSC phenotype due to donor age (32–35), sex (35, 36), tissue of origin (21, 37), and co-morbidities (38–40), as well as time spent in culture (41–45), has been extensively documented. A notable example of MSC donor-specific variance was the observation that certain bioactive secreted factors, namely tumor necrosis factor-inducible gene 6 (TSG6), show a sexual dimorphism with higher quantities secreted from female vs. males bone marrow donors (36). An increasingly important contributor to MSC phenotype is the presence of metabolic disease [obesity, type 2 diabetes, atherosclerosis, and metabolic syndrome (32, 34, 38, 39, 46–51)] within donors. A number of functional defects have been documented in adipose-derived MSCs from donors with metabolic disease, including blunted immunosuppressive potency against activated T cells (32, 38), reduced fibrinolytic activity (39, 46), and a diminished ability to halt the progression of neuroinflammation (40). In addition to the functional defect seen in MSCs isolated from patients with metabolic disease, recent work in our lab has demonstrated that healthy donor MSCs exposed to a “metabolically diseased” environment enriched in the saturated fatty acid, palmitate, are no longer suppressive, but stimulatory when cultured with activated peripheral blood mononuclear cells (52). Interestingly, MSCs from some donors were more sensitive to palmitate exposure than others, despite similar levels of potency in traditional palmitate-free co-culture assays. Collectively, this body of work highlights the need for cell manufacturing processes that consistently generate high quality MSCs and the need for *in vitro* potency assays capable of predicting MSC performance after transplant into challenging metabolic environments.

Although much of the variance in functional performance of MSCs has been attributed to intrinsic donor characteristics, only more recently has attention been focused on how specific process-related decisions may contribute to variance in MSC performance (1–3, 18, 20, 23, 25). While benefits have been reported for xeno-free systems, they have predominately been focused on improving the cell yield during manufacturing rather than the functional properties of the cells (17, 18). Additionally, the interaction of these systems with inherent donor variability is less well-understood (3, 53). Whether or not xeno-free culture systems produce MSCs that are more or less resilient for use in complex metabolic environments has yet to be determined.

In the present study, we investigated how early and late decisions regarding media supplementation modify the health and function of umbilical cord derived MSCs (ucMSCs). By growing ucMSCs from explant to experiment in one of two media supplements, either standard FBS or human platelet lysate, we were able to compare genetically identical cell preparations that had been acclimated to two different culture environments from isolation to analysis. Notably, this study design allowed us to delineate the contributions of donor intrinsic variability (nature) vs. process-derived variability (nurture). Additionally, we demonstrate for the first time that adapting an *in vitro* potency assay to mimic a disease environment can reveal the impact of process-related decisions on MSC performance within challenging metabolic environments.

## Materials and Methods

### Umbilical Cord MSC (ucMSC) Isolation

Human umbilical cords were collected through the University of Iowa Women's Health Tissue Repository's Maternal Fetal Tissue Bank with consent from the mothers (IRB#200910784). Samples were provided to the investigators in a coded fashion and this work was deemed to not be human subjects research by the University of Iowa IRB (IRB# 201708749). After delivery, samples were placed at 4°C in PBS (Biological Industries, Cat #02-023-1A-24) supplemented with 2% Penicillin-Streptomycin solution (P/S, Biological Industries, Cat #03-031-1B) and 2% Amphotericin B (AmpB, Biological Industries, Cat #03-028-1B). Umbilical cord tissue explants were plated within 48 h of delivery. To begin, the external wall of the cord was sterilized with 70% ethanol and pharmacidal spray (Biological Industries, Cat #IC-110100-05) and placed in sterile PBS. The umbilical cords were then cut into 3 mm cross-sections with a sterile razor and subdivided into 1 mm pieces. Approximately 60 cord pieces were then divided into two dry culture dishes (Corning, Cat #430599), with Wharton's Jelly placed against the plate. Umbilical cord pieces were allowed to adhere for 10–15 min before addition of media. Warm α-minimum essential medium (Biological Industries, Cat #01-042-1A) supplemented with 1% P/S, 2% AmpB, and either 10% FBS (Biological Industries, Cat #04-121-1A-US) or 5% PLTGold Human Platelet Lysate (PLT, Biological Industries, Cat #PLTGOLD100R), was then slowly added before the dishes were placed in a 37°C incubator. Media was subsequently changed every 2–3 days. After 9 days of outgrowth the cord pieces were removed and if the cells were confluent in their colonies the cells were plated as Passage 1 (P1) in T25s. If not confluent after 9 days, media was changed, and the cells were cultured an additional 3 days before plating into T25 flasks. P1 cells were grown to 80% confluence and grown for one additional passage before cryostorage using serum-free cell freezing medium (Biological Industries, Cat #05-065-1A).

### ucMSC Expansion and Growth Kinetics

ucMSCs isolated as described above were counted, plated (P1), and grown to 70–90% confluence. Cells were then harvested, counted, plated (P2), and expanded prior to cryopreservation (P3). Population doubling level at each of these splits was calculated using the formula *PDL* = *PDL*_0_+3.322(*logP*_1_−*logP*_0_), where PDL_0_ is the population doubling level at seeding, P_1_ is the total cell yield at harvest, and P_0_ is the initial cell seeding number. Cells harvested immediately after isolation were declared to have an initial PDL of 0. Time to population doubling was calculated from the initial first three cell harvests. Briefly, total cell yields at each harvest were fit using linear regression in GraphPad Prism 7 software and the linear regression equations were used to calculate the average time to population doubling for each MSC preparation.

Post-cryopreservation, MSCs were thawed at 37°C until ~1 mm^3^ piece of ice remained. The cells were then transferred immediately into the appropriate pre-warmed media condition and allowed to attach overnight. Media was switched to remove cryopreservation media and cells were grown out to 70–90% confluence. Population doublings after cryostorage were calculated as before from the total cell yields after each cell harvest to confirm consistency of growth kinetics in the medias before and after cryopreservation.

### BSA Preparation and Palmitate-BSA Conjugation

Bovine serum albumin (BSA) and palmitate conjugated to BSA were prepared according to a previously described protocol (52, 54, 55). Briefly, to prepare the BSA solution, 20% (w/v) fatty acid free bovine albumin (MP Biomedicals, Cat #0219477205) was gradually added to Ultra Pure water (Biological Industries, Cat #01-866-1A) at 52°C with gentle agitation until the BSA was fully dissolved, yielding a transparent yellow solution. To prepare a palmitate-BSA solution, 2.6% (w/v) of palmitic acid (Sigma-Aldrich, Cat #P0500) was added to 0.1 M NaOH (Sigma-Aldrich, Cat #221465) and heated to 70°C until fully dissolved, yielding a concentration of 100 mM. To conjugate palmitate to BSA, the 100 mM stock of palmitate in 0.1 M NaOH was added to 20% BSA at a ratio of 1:9 and heated at 55°C for 10 min. After conjugation, both the 20% (w/v) BSA solution and 10 mM Palmitate-BSA solutions were filter sterilized using a 0.45 μm PES filter (Celltreat, Cat #229709). Solutions were then aliquoted and stored at −20°C. Before use, solutions were warmed to 37°C. The total amount of BSA was held constant in all conditions by mixing ratios of fatty acid free BSA to palmitate-BSA as follows: BSA only (4:0), 0.1 mM palmitate-BSA (3:1), 0.2 mM palmitate-BSA (2:2), and 0.4 mM palmitate-BSA (0:4).

### ucMSC Validation (Immunophenotyping and Differentiation)

To validate ucMSC identity via immunophenotyping, cells were stained to confirm CD105, CD73, and CD90 expression and the absence of CD45, CD34, CD11b, CD19, and HLA-DR as defined by the MSC minimal criteria (56). Positive markers were confirmed using FITC-CD105 antibody (BD Biosciences, Cat #561443), PE.Cy7-CD73 antibody (BD Biosciences, Cat #561258), and PE-CD90 antibody (BD Biosciences, Cat #A15794) with appropriate isotype controls [FITC Mouse lgG1k (BD Biosciences, Cat #556649), PE.Cy7 Mouse lgG1k (BD Biosciences, Cat #557872), and PE-CD90 Mouse lgG1 (Invitrogen, Cat #GM4993)]. A negative marker cocktail and isotype staining was performed using the PE hMSC Negative Cocktail (BD Biosciences, Cat #562530), as per manufacturer's instructions.

For trilineage differentiation (56), each preparation of ucMSCs (seven donors, maintained in either FBS-media or PLT-media) was differentiated to osteoblasts, chondroblasts, and adipocytes by culturing in osteogenic (Biological Industries, Cat #05-440-1B), chondrogenic (Biological Industries, Cat #05-220-1B-KT), or adipogenic (Biological Industries, Cat #05-330-1B-KT) differentiation medias, respectively. Briefly, for osteogenic and adipogenic differentiation, ucMSCs were plated at a density of 60,000 cells/well in 24-well plates and grown in either FBS- or PLT-media for 24 h. Cells were then visualized to ensure >80% confluence and media was exchanged to osteogenic or adipogenic differentiation medias. Cultures were maintained for 15–18 days with media exchanges every 3–4 days. Osteogenic lineage was confirmed using both bright-field imaging and Alizarin Red for mineral deposition. For adipogenic lineage confirmation, lipid droplets were identified via AdipoRed staining and fluorescent microscopy. For chondrogenic differentiation, a micromass culture (10 uL of 10 million/mL ucMSCs in chondrogenic differentiation media) was incubated for 2 h to allow for spontaneous spheroid formation in 96-well spheroid plates (Corning, Cat #4515). After 2 h, 200 uL of chondrogenic media was added for subsequent culture. Spheroid cultures were maintained for 18 days with media exchanges occurring every 3–4 days. On Day 18, chondrogenic spheroids were fixed for 5 min with 10% Formalin then mounted in Optimal Cutting Temperature (OCT) Compound. Sectioning of the chondrogenic pellets was performed by the University of Iowa Comparative Pathology Core and tissues were subsequently stained with Safranin O.

### Metabolic Health and Viability Assays

To assess the metabolic function of ucMSCs exposed to palmitate, we performed an XTT assay (Cell Proliferation Kit-XTT based, Biological Industries, Cat #20-300-1000). For each media condition, 1,000 ucMSCs from every donor were plated into a 96-well plate. All wells had a total media volume of 100 μL. The ucMSCs were treated with 20% (w/v) BSA or 0.1, 0.2, or 0.4-mM palmitate-BSA as described above. After 96 h, Activation Reagent was added to warmed XTT Reagent at a ratio of 1:5,000 and 50 μL of XTT solution was added to the media of each well. The plate was read at 475 and 660 nm absorbance at 1 and 2 h time points by a microplate reader. The background reading was subtracted for all wells, and within each media condition, the background-subtracted reading was normalized to a media-only control.

To assess changes in viability, ucMSCs were incubated at 37°C with BSA, 0.1, 0.2, or 0.4 mM Palmitate-BSA for 96 h. Both detached cells in the media and adherent cells were collected and pooled for analysis. Cells were pelleted, resuspended in prepared Annexin/PI binding buffer [20 mM HEPES at pH 7.4 (Invitrogen, Cat #A14291DJ), 150 mM sodium chloride (American Bio, Cat #AB01915-10000), and 2.5 mM calcium chloride (Sigma Aldrich, Cat #C8106-500g)], and stained with 90 μg/mL FITC-Annexin V (Biolegend, Cat #640945 and 640906) and 1 mg/mL propidium iodide (Invitrogen, Cat #P3566) to final concentrations of 4.5 and 50 μg/mL, respectively, in Annexin/PI binding buffer. Samples were incubated for 30 min in the dark at room temperature. Samples were then diluted with Annexin/PI binding buffer and analyzed via flow cytometry (Accuri C6, BD Biosciences).

### MSC:PBMC Co-culture With Palmitate Challenge

Peripheral blood mononuclear cells (PBMCs) from a de-identified donor were isolated from a leukapheresis reduction cone made available by the DeGowin Blood Center at the University of Iowa Hospitals and Clinics. After isolation, PBMCs were cryopreserved in a solution consisting of 50% RPMI (ThermoFisher, Cat #11875093), 40% FBS (VWR, Cat #97068085), and 10% DMSO (Fisher Scientific, Cat #D128) until use. PBMCs were thawed at 37°C 1 h prior to staining and plated in RPMI containing 10% FBS, 1% L-glutamine , and 1% P/S. In order to track generational proliferation of the PBMCs, PBMCs were stained with CFSE Cell Division Tracker Kit (Biolegend, Cat #423801) according to the manufacturer's protocol. Briefly, PBMCs resuspended in PBS at 1 million cells per mL were stained with 2 μL of 10 mM CFSE per 10 million cells to yield a final CFSE concentration of 1 μM. PBMCs were incubated at 37°C for 15 min, centrifuged at 500 g for 8 min, resuspended in RPMI to neutralize the dye, and incubated at 37°C for 30 min. Cells were centrifuged again, resuspended at 1 million per mL, and stored at 37°C until plating with the MSCs.

All 14 preparations of ucMSCs were harvested after staining the PBMCs, and 50,000 cells were plated into 24-well plates. MSCs were allowed to attach for 1 h. Prior to plating, CFSE stained PBMCs were mixed 1:1 with Human CD3/CD28 Dynabeads (Thermo Fisher, Cat #11132D) to activate T cells within the mixed population. 200,000 stimulated PBMCs were added to each MSC condition to yield an MSC to PBMC ratio of 1:4. Total media volume in each well was set at 750 μL and all co-culture conditions were either treated with BSA alone, or 0.1, 0.2, or 0.4 mM palmitate-BSA to simulate metabolic stress. PBMCs with or without Dynabeads were plated as positive and negative controls for PBMC activation and proliferation. After 5 days, PBMCs were isolated by collecting the non-adherent cell fraction from the plates. These cells were then centrifuged, the supernatants collected, and resuspended in RPMI. Supernatants were stored at −20°C for future analysis of Granzyme-B levels using a bead-based ELISA per the manufacturer's instructions (BD Biosciences, Cat #560304). PBMC proliferation was analyzed by flow cytometry (Accuri C6, BD Biosciences).

### Media Exchange Experiments

Two vials of P4 ucMSC from donor 4600 isolated with FBS media, were thawed and plated in either FBS (FBS-FBS) or PLT (FBS-PLT) supplemented media. Likewise, two vials of P4 ucMSC from donor 4600 were thawed and plated in either FBS (PLT-FBS) or PLT (PLT-PLT) supplemented media. After culturing for 72 h, the ucMSCs were counted and replated to assess their growth kinetics. The cells were then cultured for another passage (96 h), lifted, counted, and plated for either co-culture or imaging experiments. For co-culture with PBMCs, each preparation of ucMSCs were plated at a ratio 1 MSC: 4 PBMCs. PBMCs were stained with CFSE and stimulated with Dynabeads, as previously described. The co-culture was incubated for 5 days, followed by flow cytometry analysis. For morphological analysis, ucMSCs were plated at 1,000 ucMSCs/cm2 in 6-well plate and cultured for an additional 48 h. Cells were then fixed for 5 min (10% neutral buffered Formalin, Sigma-Aldrich, Cat# HT501128-4L), followed by permeabilization for 5 min (0.1% v/v Triton® X-100 in PBS, Sigma-Aldrich, Cat#T9284-500 mL), at room temperature. After permeabilization, the cells were stained with ActinRed 555 ReadyProbes Reagent (Cat# R37112, Thermo Fisher Scientific) according to the manufacturer's protocol. Briefly, for every 1 milliliter of PBS, 2 drops of reagent were added. The cells were then incubated with 1 mL of reagent in PBS at 37°C for 30 min. During the last 10 min of ActinRed staining, 1 ug/mL of Hoechst 33342 (Cat# H3570, Invitrogen) was added. Cells were then imaged using a fluorescent microscope (Leica DMI6000) at 20× magnification.

### Statistical Analysis

Statistical analysis was pre-planned prior to collection of data and performed within GraphPad Prism 7. For all assays involving multiple donors, each donor was considered an independent “n.” For assays involving a single donor, independent replicate experiments were considered as “n.” Specific statistical tests used for each data set is listed in the caption of each figure.

## Results

### PLT-Media Enhances ucMSC Growth Kinetics and Overall Cell Yield Compared to FBS-Media

**Figure 1:**
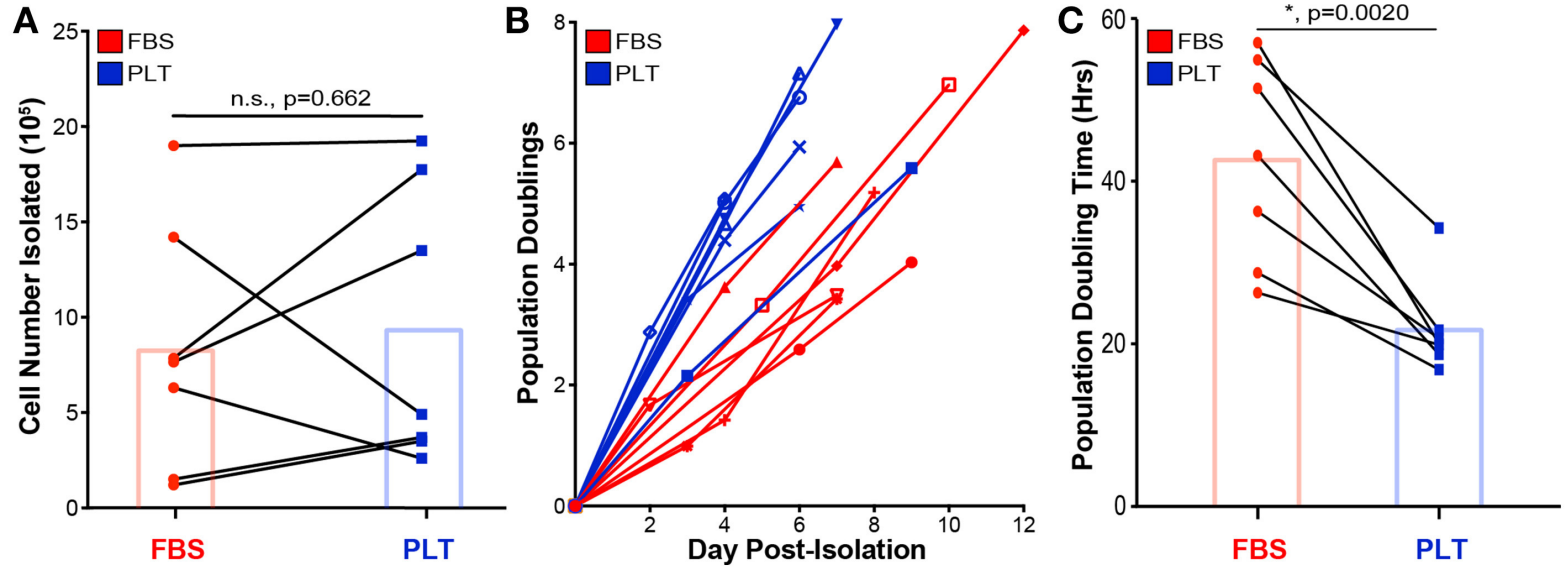
PLT-media accelerates ucMSC growth kinetics and overall cell yield but not initial cell isolation from tissue explant. **(A)** Total cell number isolated from the initial umbilical cord tissue explant was determined via hemacytometer after 9–12 days of outgrowth. Donor paired ucMSC preparations [single donor grown in either FBS-media (red circle) or PLT-media (blue square)] are represented by connected lines. Bar graph displays the mean for all donors (paired *t*-test, *n* = 7 ucMSC donors, *n.s., p* = *0.662*). **(B)** Cells isolated from the original tissue explant were plated at Day 0 Post-Isolation (P1) and allowed to grow until 70–80% confluence, before passaging. Each line represents a different preparation with each node representing the cumulative population doublings determined at P1, P2, and P3. **(C)** An average population doubling time was determined via linear regression from P1 to P3 outgrowth data for each donor. Donor paired ucMSC preparations are shown for FBS-media (red circle) and PLT-media (blue square). Bar graph displays the mean value across all donors (paired *t*-test, *n* = 7 ucMSC donors, *, *p* = *0.0020*).

To better understand how early choices in culture isolation and expansion affect growth rates and yield of ucMSCs, equivalent amounts of umbilical cord explants from the same donor were plated into two different media formulations (*n* = 7 umbilical cord donors), one supplemented with xeno-free 5% PLT (PLT-media) and one supplemented with traditional 10% FBS (FBS-media). Umbilical cord tissue explants were incubated to allow for ucMSC migration out of the cord and onto the underlying tissue culture plastic. After 11 days, ucMSCs were counted to determine initial cell yield (Figure 1A) and then culture-expanded for two additional passages prior to cryopreservation for subsequent experiments. Growing umbilical cord tissue explants in PLT-media did not enhance the initial amount of ucMSCs isolated (Figure 1A). Though the initial cell yield was not different between media preparations, subsequent outgrowth of ucMSCs in PLT-media resulted in a higher yield of cells (Figure 1B) and a significantly lower population doubling time compared to FBS-media (Figure 1C). To ensure that there were not large differences in total population doublings between the two different media preparations, ucMSCs were cryopreserved once 70–80% confluence was reached leading to an equivalent number of total population doublings prior to cryopreservation (Supplemental Figure 1). Additionally, all ucMSC preparations were characterized and found to meet ISCT minimal criteria for classification as MSCs (56), including positive staining for CD105, CD73, and CD90 and negative staining for CD45, CD34, CD19, CD11b, and HLA-DR, as well as trilineage differentiation potential (Supplemental Figures 2–4).

### Media Supplementation Dictates a Differential Response to Palmitate-Induced Metabolic Stress and Apoptosis

**Figure 2:**
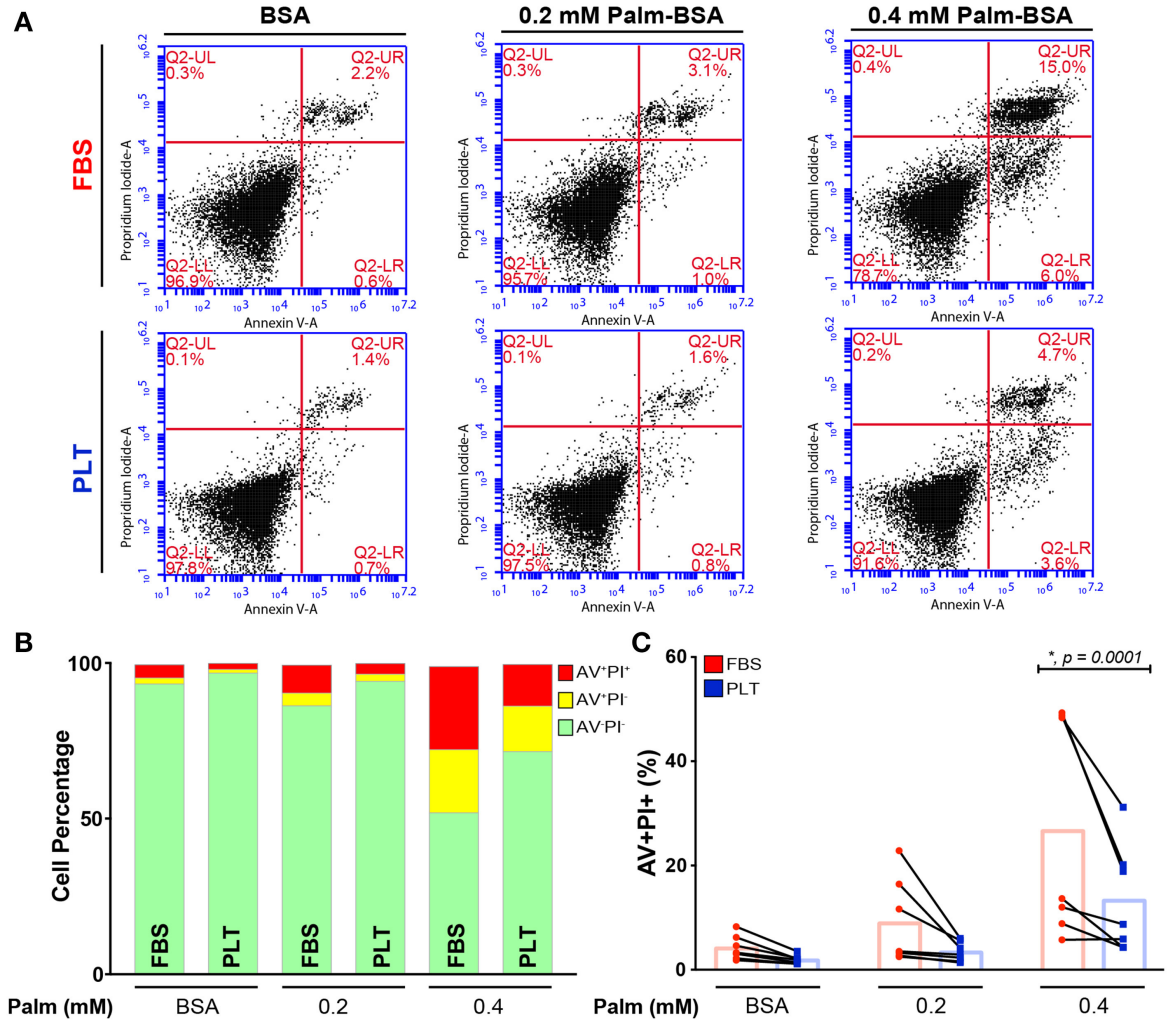
ucMSCs are largely protected from the pro-apoptotic effects of palmitate exposure in PLT-media. **(A)** Representative flow cytometry dot plots from a single donor (3004iA) showing Annexin V (AV, x-axis) and Propidium Iodide (PI, y-axis) staining after 96 h of palmitate exposure. Top row shows the FBS-ucMSC preparation; bottom row shows the PLT-ucMSC preparation. **(B)** Summary data from FBS-ucMSC and PLT-ucMSC preparations showing average percent of viable (AV^−^PI^−^, green), early apoptotic (AV^+^PI^−^, yellow), and late apoptotic (AV^+^PI^+^, red) ucMSCs after 96 h of palmitate exposure. **(C)** Percent of late apoptotic cells (AV^+^PI^+^, red) at each concentration of palmitate. Donor paired ucMSC preparations are connected by a line between FBS-ucMSC (red circle) and PLT-ucMSC (blue square) preparation. Bar graphs represent the mean value across all donors (2-way ANOVA with Sidak Correction for multiple comparisons to FBS-ucMSC, *n* = 7 ucMSC donors, BSA: *n.s., p* = *0.6518*, 0.2: *n.s., p* = *0.0621*, 0.4: *, p = *0.0001*).

In our previous study, we found that increased levels of palmitate exposure led to a slight increase in levels of apoptosis and a significant decrease in the metabolic activity of bone marrow-derived MSCs (as determined by NAD^+^/NADH ratio) (52). To determine whether media supplementation led to differences in palmitate-induced apoptosis, we cultured ucMSCs in FBS- or PLT-media and exposed the cells to a range of physiological doses (57, 58) of palmitate for 96 h. Across all palmitate conditions, there was a larger overall increase in the proportion of both early (Annexin V+/Propidium Iodide-, AV+/PI-) and late (AV+/PI+) stage apoptotic cells in FBS-media (Figures 2A,B) compared to PLT-media. Notably, in FBS-media, ucMSC preparations showed a 2.0-fold increase in susceptibility to palmitate-induced apoptosis compared to paired PLT-media preparations as measured by late-stage apoptotic cells (Figure 2C). Less overall apoptosis was evident in all PLT-media donors across all tested conditions with FBS-media donors showing 2.2 and 2.6 times as many AV+/PI+ cells after 96 h of BSA and 0.2 mM Palm-BSA exposure, respectively; however, only 0.4 mM Palm-BSA achieved a significant difference in the number of late-stage apoptotic cells.

**Figure 3:**
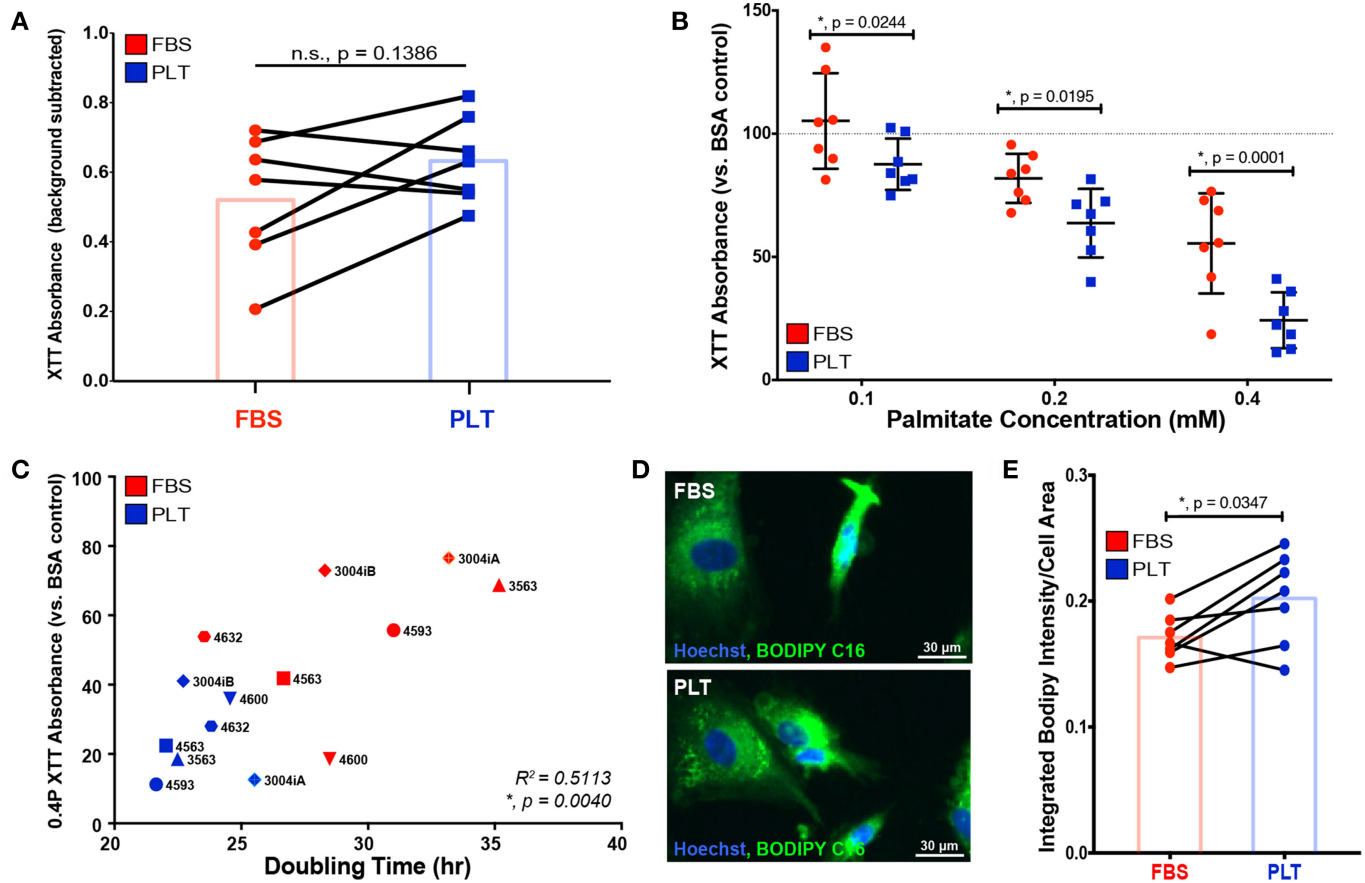
ucMSCs grown in PLT-media show decreased overall metabolic health after palmitate exposure which correlates with higher overall proliferative rates. **(A)** Basal NAD^+^/NADH ratio was determined using XTT assay after 96 h of exposure to vehicle control (BSA) in all FBS-ucMSC and PLT-ucMSC preparations (absorbance value = total absorbance – background absorbance from media only control). Paired ucMSC preparations are connected by a line between FBS-ucMSC (red circle) and PLT-ucMSC (blue square) preparations. Bar graphs represent the mean value across all donors (paired *t*-test, *n* = 7 ucMSC donors, *n.s., p* = *0.1386*). **(B)** The relative NAD^+^/NADH ratio after 96 h of palmitate exposure was determined by normalizing the XTT absorbance to the within donor BSA control for each ucMSC preparation (BSA value is represented by the dotted line). FBS-ucMSCs (red circle) were normalized to BSA in FBS-media and PLT-ucMSCs (blue square) were normalized to BSA in PLT-media (2-way ANOVA with Sidak Correction for multiple comparisons to FBS-ucMSC, *n* = 7 ucMSC donors, 0.1: *, p = *0.0244*, 0.2: *, p = *0.0195*, 0.4: *, p = *0.0001*). **(C)** Correlation plot comparing XTT absorbance at 0.4 mM Palm-BSA (y-axis) to population doubling time in the absence of palmitate (x-axis) for each donor preparation (Pearson correlation coefficient, *n* = 14 ucMSC preparations, R^2^ = 0.5113, *, p = *0.0040*). **(D)** Representative images of FBS-ucMSC and PLT-ucMSC preparations incubated with the fluorescent palmitate analog, BODIPY FL C16, and counterstained with Hoechst 33342 for nuclear identification, 20× magnification, Scale = 30 μm. **(E)** The total integrated intensity of fluorescent BODIPY FL C16 and cell area were determined via CellProfiler quantification of the images represented in **(D)**. Donor paired ucMSC preparations are connected by a line between FBS-ucMSC (red circle) and PLT-ucMSC (blue square) preparations. Bar graphs represent the mean value across all donors (paired *t*-test, *n* = 7 ucMSC donors, *, p = *0.0347*).

Next, to determine the effect of palmitate on the metabolic health of the ucMSCs donors, we cultured ucMSCs in increasing levels of palmitate and assessed NAD^+^/NADH ratio, i.e., the redox state of the cells, by XTT. Treatment with the vehicle control (BSA) showed no difference in the baseline NAD^+^/NADH ratio; however, the mean NAD^+^/NADH ratio was higher in PLT-media compared to FBS-media (Figure 3A), which is consistent with the faster overall growth kinetics in PLT-media observed previously (Figure 1C). Interestingly, though FBS-media preparations showed a greater overall susceptibility to palmitate-induced apoptosis, by XTT assay, PLT-media preparations were affected more by the presence of palmitate at every dose tested (Figure 3B). Given that NAD^+^/NADH ratio is a metric of the redox state of the cell which includes both metabolic health and proliferative ability (59), we compared the doubling time of each preparation previously calculated to the XTT absorbance at the highest dose of palmitate tested (0.4 mM Palm-BSA). As expected, there was a significant positive correlation (R^2^ = 0.5113, ^*^, *p* = 0.0040, Pearson correlation coefficient) between the doubling time of each preparation and the decline in XTT absorbance observed upon palmitate exposure, with lower doubling times associated with a larger decline in NAD^+^/NADH ratios upon palmitate exposure (Figure 3C).

In addition to altering cellular metabolism, proliferating cells also increase uptake of fatty acids to produce cellular membranes (60). To determine whether the higher proliferative rate of PLT-media donors led to increased uptake of exogenous fatty acids, all 14 ucMSC preparations were incubated with a fluorescent palmitate analog, BODIPY FL C16, and imaged to quantify BODIPY uptake on a per cell basis. The total integrated intensity of the BODIPY signal was quantified using the image processing software, CellProfiler. The total BODIPY signal was then divided by the overall cell area to account for differences in size between ucMSC grown in FBS- vs. PLT-media. All ucMSC preparations showed uptake of BODIPY FL C16 (Figure 3D), but the overall uptake was increased in ucMSC grown in PLT-media compared to those grown in FBS-media for nearly every donor (Figure 3E, *FBS-media mean* = *0.1710* ±*0.0181 vs. PLT-media mean* = *0.2019* ± *0.0365*), suggesting that PLT-media grown ucMSC preparations increase their uptake of exogenous palmitate from the environment compared to FBS-media preparations.

### Immunosuppressive Potency of ucMSCs Is Critically Dependent on Media Supplementation

In our previous study, we discovered that palmitate exposure converted FBS-cultured bone marrow derived MSC from an immunosuppressive to an immunostimulatory profile in co-culture with stimulated T cells (52). Given the broad range of potential alterations precipitated by culturing ucMSCs in FBS- compared to PLT-supplemented media (10, 30, 61), we sought to determine whether culturing ucMSCs in a xeno-free, PLT based culture system would rescue the functional immunosuppressive defect previously seen in co-culture after palmitate exposure. For this study, we used a single PBMC donor in order to focus solely on the contribution of media supplementation on ucMSC potency within PBMC co-cultures.

**Figure 4:**
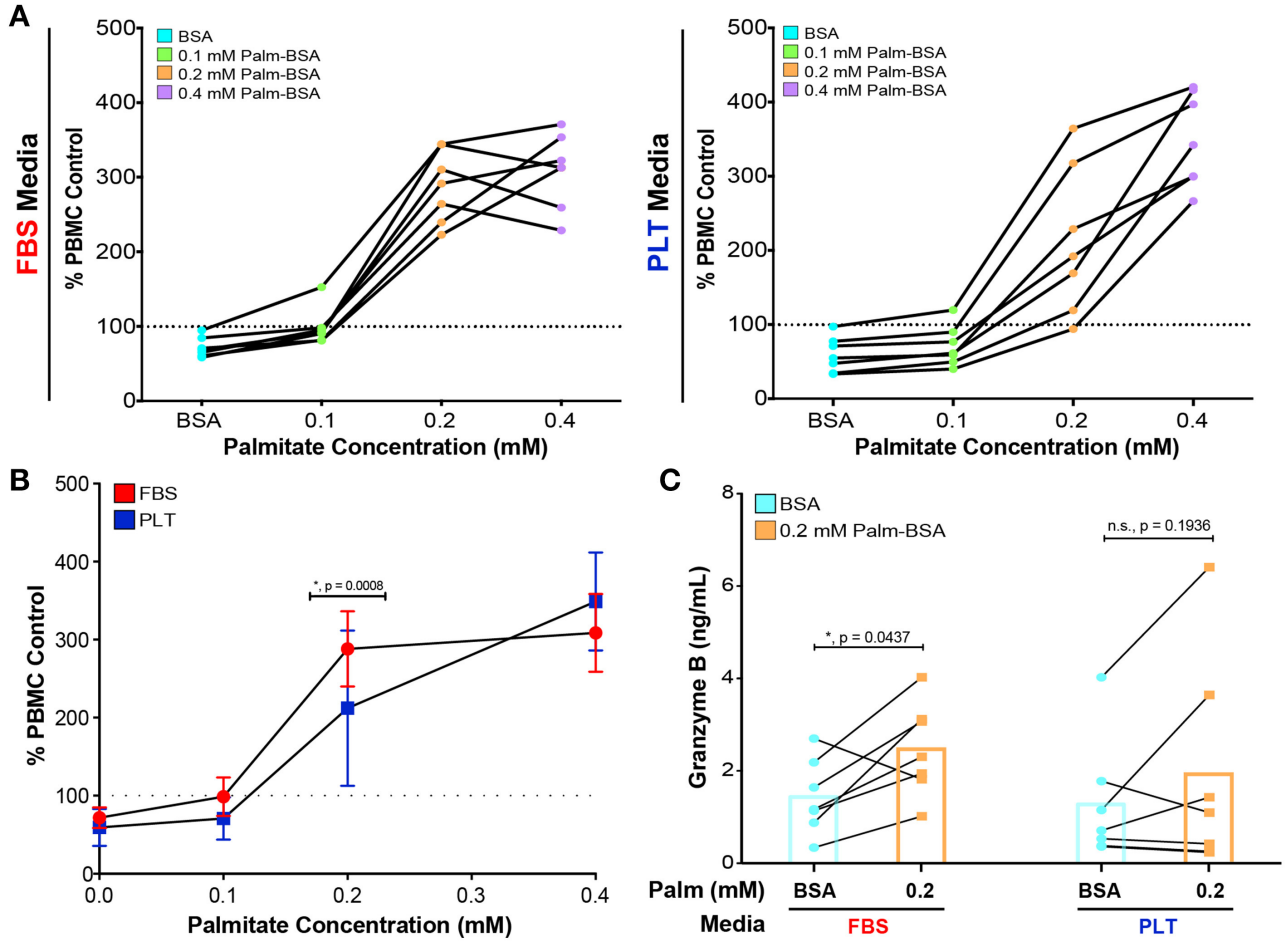
PLT-media decreases the severity of palmitate-induced decline in immunosuppressive potency compared to FBS-media. **(A)** PBMCs, stimulated with CD3/CD28 and stained with CFSE, were exposed to increasing concentrations of palmitate in the absence (PBMC control) or presence of MSCs (1:4 ratio of MSC:PBMC). All data points in **(A)** are displayed relative to the proliferation in the PBMC control at that palmitate exposure (dotted line on graph). Each solid line represents the dose response of an individual donor, with FBS-uMSC donors (left) and PLT-ucMSC donors (right). Data points below the dotted line are immunosuppressive and data points above the line are immunostimulatory. A single PBMC donor was used. **(B)** Summary data demonstrating the dose response of all FBS-ucMSC (red) and PLT-ucMSC (blue) donors [mean ± SD, *n* = 7 ucMSC donors, 2-way ANOVA with Sidak correction for multiple comparisons to FBS-ucMSC, 0 (BSA): *n.s., p* = *0.9111*, 0.1: *n.s., p* = *0.3560*, 0.2: *, p = *0.0008*, 0.4: *n.s., p* = *0.0914*]. **(C)** Protein levels of Granzyme B within the supernatant of co-cultures as determined by bead-based ELISA assay. Paired values connected by a solid line represent a single donor exposed to vehicle control (blue circles) or 0.2 mM palmitate (orange square). Bar graphs represent the mean value across all donors (2-way ANOVA with Sidak correction for multiple comparisons to BSA control, FBS: *, p = *0.0437*, PLT: *n.s., p* = *0.1936*).

Notably, all ucMSC preparations, regardless of previous growth in FBS- or PLT-media, suppressed the proliferation of activated PBMCs (Figure 4A, data points below the dotted line are immunosuppressive). Both FBS- and PLT-media preparations showed a large variation in baseline immunosuppressive potency, suppressing T cell proliferation by 5–42% in FBS-media and 3–66% in PLT-media. On average, PLT-media preparations were more suppressive at baseline (*FBS-media BSA mean* = *71* ±*13, PLT-media BSA mean* = *59* ± *23*); however, due to the large amount of donor-to-donor variation this value did not reach statistical significance. Interestingly, although PLT-media preparations showed less variation in growth kinetics, palmitate-induced apoptosis, and changes in NAD^+^/NADH, they had a wider variation in baseline immunosuppressive ability compared to FBS counterparts. At low physiological levels of palmitate (0.1 mM Palm-BSA), FBS-media preparations showed a greater decline in immunosuppressive potency compared to PLT-media preparations. Transitioning from BSA to 0.1 mM Palm-BSA resulted in a more rapid loss of PBMC suppression for FBS-media MSC preparations compared to PLT-media MSC preparations (27 ± 16% increase in PBMC proliferation for FBS vs. 11 ± 6.5% increase for PLT, Figure 4B). At the extreme 0.4 mM Palm-BSA dose, both FBS and PLT-media preparations showed a strong immunostimulatory profile but differences between the groups did not reach statistical significance (*95% CI of difference:* −*85% to* +*4%, p* = *0.09*). At 0.2 mM Palm-BSA, a level closer to the average serum level in patients with obesity and/or type 2 diabetes (57, 62), all FBS-media preparations and all but one PLT-media preparation converted to an immunostimulatory phenotype, which is consistent with our previously published results. However, PLT-media preparations showed a significantly less severe immunostimulatory profile compared to FBS-media preparations (Figure 4B, *FBS-media 0.2 Palm-BSA mean* = *288 vs. PLT-media 0.2 Palm-BSA, mean* = *212, 95% CI of difference: 31% to 120%, p* = *0.0008*). Growth in PLT-media, therefore, appears to protect ucMSC immunomodulatory potential from the full extent of damage inflicted by exposure to low to moderate physiological doses of palmitate.

In order to better understand the role of the PBMCs within the co-culture system, supernatants from all of the co-cultures were collected and analyzed for Granzyme B (GRZ-B), an immune effector molecule commonly secreted by cytotoxic T cells and NK cells (63). In agreement with the PBMC proliferation data (Figures 4A,B), palmitate exposure led to a significant increase in GRZ-B in co-cultures with FBS-ucMSC, while co-cultures with PLT-ucMSCs only showed a slight non-significant increase (Figure 4C, Supplemental Table 1). Interestingly, this inflammatory profile seems to be dependent on both specific ucMSC donor characteristics, as well as the media supplementation used during cell expansion. In some donors, for example donor 3004iA, the preparation in FBS showed a similar change in PBMC proliferation and GRZ-B secretion in response to palmitate as the PLT preparation (FBS: 152%, 806 pg/mL GRZ-B vs. PLT: 174%, 719 pg/mL GRZ-B, Supplemental Table 1). However, with donor 4600, media supplementation greatly affected the performance of the donor in co-culture, with the FBS preparation showing 2.69 times higher PBMC proliferation and 18.33 times higher GRZ-B levels than the PLT preparation (Supplemental Table 1). This data, therefore, suggests that certain donors may be more highly affected by the choice of media supplementation during culture expansion, while other donors may behave the same regardless of choice of media supplementation.

### Changing Media Supplementation Greatly Alters the Growth Kinetics of ucMSCs

**Figure 5:**
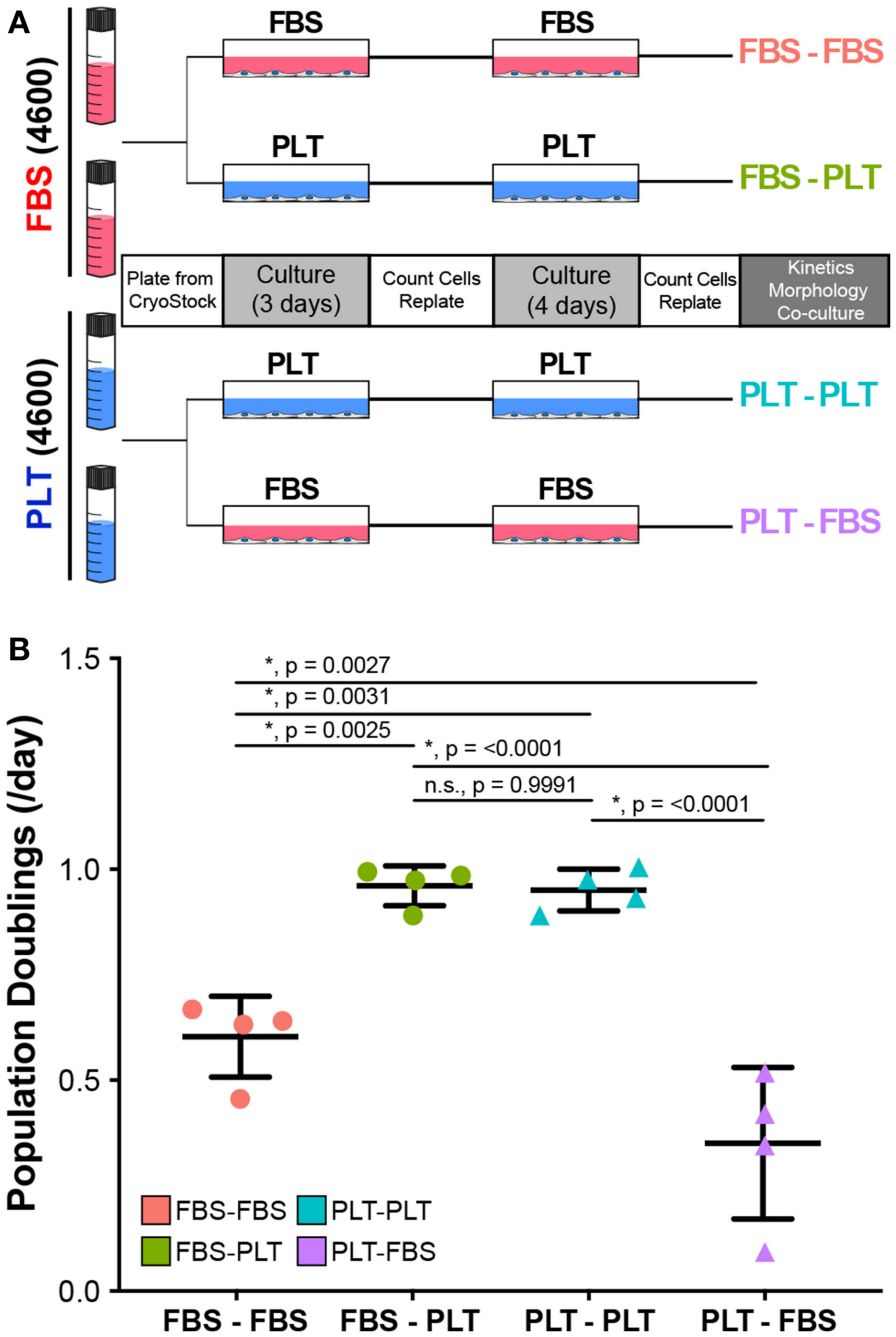
Growth kinetics in ucMSCs are significantly altered by changes in media supplementation. **(A)** Schematic of workflow for media exchange experiments. **(B)** Daily population doublings were calculated after 7 days in maintenance media (FBS-FBS, PLT-PLT) or transition media (FBS-PLT, PLT-FBS) (1-way ANOVA, *n* = 4 independent experiments, * denotes significance *p* < 0.05). Circles represent ucMSC preparations that were initially isolated and expanded in FBS-media and triangles represent preparations originally isolated and expanded in PLT-media.

In the previous series of experiments analyzing how media supplementation affected MSC immunosuppressive ability in harsh metabolic environments, we observed that there were several donors (4600, 3004iB, and 4563) that had a large change in PBMC suppression depending on their growth in FBS or PLT supplemented medias (Supplemental Table 1). These donors, therefore, derived the greatest benefit from isolation and expansion in PLT-media. We, therefore, sought to determine if the cells retained a “memory” of the early isolation environment or if changing the culture environment could alter the cells' behavior. To this end, we grew FBS-preparations of donor 4600 in FBS (FBS-FBS) or switched the media to PLT (FBS-PLT) and grew PLT-preparations of 4600 in PLT (PLT-PLT) or switched the media to FBS (PLT-FBS) (Figure 5A). In order to acclimate the cells to the new media supplementation and control for passage effects, we cultured each preparation for 7 days with one passage event prior to analysis.

Interestingly, we found that switching the media supplementation had drastic effects on the growth kinetics of the preparation. Preparations of donor 4600 that were maintained in their original media supplementation (FBS-FBS and PLT-PLT) had growth kinetics (Figure 5B) similar to those seen at earlier passages (Figure 1C). Notably, switching a FBS-preparation into PLT-media greatly improved the growth kinetics, with FBS-PLT cells growing at an average rate of 0.96 population doublings/day, a rate more similar to PLT-PLT preparations than FBS-FBS preparations (Figure 5B). Surprisingly, switching a PLT-preparation into FBS-media drastically reduced the proliferative ability of the cells resulting in an average growth rate of just 0.34 population doublings/day, a rate lower than cells maintained in FBS alone (Figure 5B). Given that the data represents growth rates after one passage and 7 total days in culture, a growth halt due to shock or adaptation delay alone does not explain the drastic difference between PLT preparations maintained in PLT compared to those same preparations transitioned to FBS-media.

### Changes in Media Supplementation Drive Alterations in Cytoplasmic and Nuclear Morphological Features of ucMSCs

**Figure 6:**
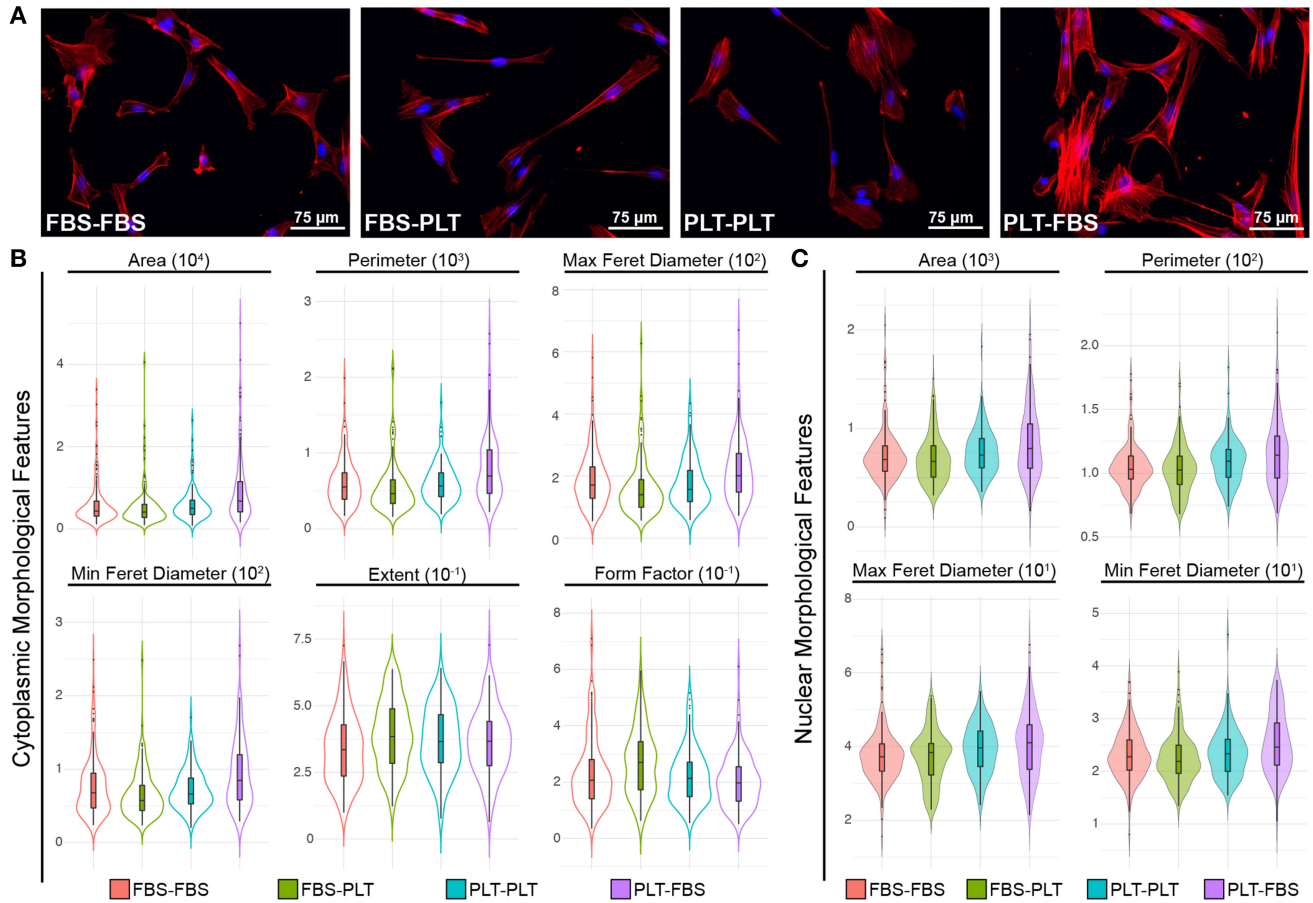
ucMSCs respond to changes in media supplementation by modifying cytoplasmic and nuclear morphological features. **(A)** Representative images of nuclear (Hoechst 3342, blue) and cytoplasmic (ActinRed 555, red) features after 7 days of maintenance (FBS-FBS, PLT-PLT) or transition media (FBS-PLT, PLT-FBS). Each image was acquired at 20× magnification, Scale = 30 μm. **(B)** Single-cell cytoplasmic morphological features were plotted as violin plots with embedded box-plots (box: 25th, 50th, and 75th percentiles; whiskers: 10th and 90th percentiles). Only six features that were found to be significantly different between groups by 1-way ANOVA (refer to Supplemental Table 2 for values) are represented. Approximately 10–20 whole cells were detected per image in 10 images for each media condition, yielding ~150–200 cells per media condition. **(C)** Single-cell nuclear morphological features were plotted as violin plots with embedded box-plots (10th, 25th, 50th, 75th, and 90th percentiles). Four nuclear features showed statistically significant differences between groups by 1-way ANOVA (refer to Supplemental Table 2 for values).

Recently, several groups have demonstrated the utility of analyzing complex morphological features of MSCs to demonstrate phenotypic changes precipitated by changes in priming conditions or media supplementation (10, 64–66). During the initial outgrowth of ucMSCs in new media supplementation, it appeared that ucMSCs took on the morphological features of the new media (i.e., FBS-PLT looked more similar to PLT-PLT than FBS-FBS preparations). To quantify this morphological transition, we fixed and stained cells from each of the media conditions (FBS-FBS, FBS-PLT, PLT-PLT, and PLT-FBS) with an F-actin stain to delineate the cytoplasm and Hoechst 33342 to define the nucleus (Figure 6A). We then analyzed the features of cells in each media condition with a modified pipeline in CellProfiler (64). Only cytoplasmic or nuclear features that showed statistically significant differences between media groups by one-way ANOVA were pursued (Figure 6B, Supplemental Table 2).

Interestingly, in line with the growth kinetic changes, the PLT-FBS transition led to the highest number of morphological changes when compared to any other media combination (Figure 6B). In FBS-media, PLT cells showed increased area, larger maximum and minimum feret diameters, and a larger overall perimeter in both the cytoplasm and nucleus. Although originally under bright field, FBS and PLT preparations appeared different, there were no cytoplasmic features that showed a statistically significant difference between FBS-FBS and PLT-PLT. However, at the nuclear level, PLT-PLT cells had a larger overall maximum and minimum feret diameter, as well as higher mean nuclear area and perimeter values (though area and perimeter were not statistically significant). These nuclear changes in PLT-PLT cells may be representative of more cells in S-phase of the cell cycle due to the higher overall growth rate observed (52, 67). However, PLT-FBS cells also showed a larger maximum and minimum feret diameter, area, and perimeter within the nucleus, while the overall growth rate within these cells is highly reduced compared to any other media combination. Though it would seem logical that FBS-PLT cells might take on a morphological profile between FBS-FBS and PLT-PLT cells (10), the morphological profile of FBS-PLT cells was distinct from either of the other preparations. At the cytoplasmic level, FBS-PLT cells were of a similar area, perimeter, and max/min feret diameter compared to both FBS-FBS and PLT-PLT cells; however, FBS-PLT cells showed a more rounded or spherical morphology than FBS-FBS and PLT-PLT cells with a higher extent and form factor measurement. Notably, however, at the nuclear level the mean values of most FBS-PLT measures were more similar to those of the FBS-FBS preparations, while the mean values of most PLT-FBS measures were more similar to those of the PLT-PLT cells (Supplemental Table 2). Cytoplasmic and nuclear changes may then be differentially regulated by transitions into new media supplementation, with cytoplasmic features being more readily plastic than their nuclear counterparts.

### PLT-Media Improves Functional Resiliency in Challenging Metabolic Environments

Finally, we wanted to determine if transitioning media supplementation from FBS to PLT could effectively rescue the function of an ucMSC donor that had performed poorly in a co-culture potency assay when challenged with palmitate. To test the ability of media supplementation to effectively rescue a “poor performing donor,” we used our previous culture scheme (Figure 6A) to prepare four different ucMSC preparations from the same donor (4600). These preparations of ucMSCs were then plated with activated PBMCs from three independent donors to test for immunosuppressive potency in the presence or absence of palmitate. Given the significant difference in functional performance between FBS-media and PLT-media donors previously observed in co-culture with 0.2 mM palmitate (Figure 4B, Supplemental Table 1), we chose this dose for the palmitate exposure condition.

**Figure 7:**
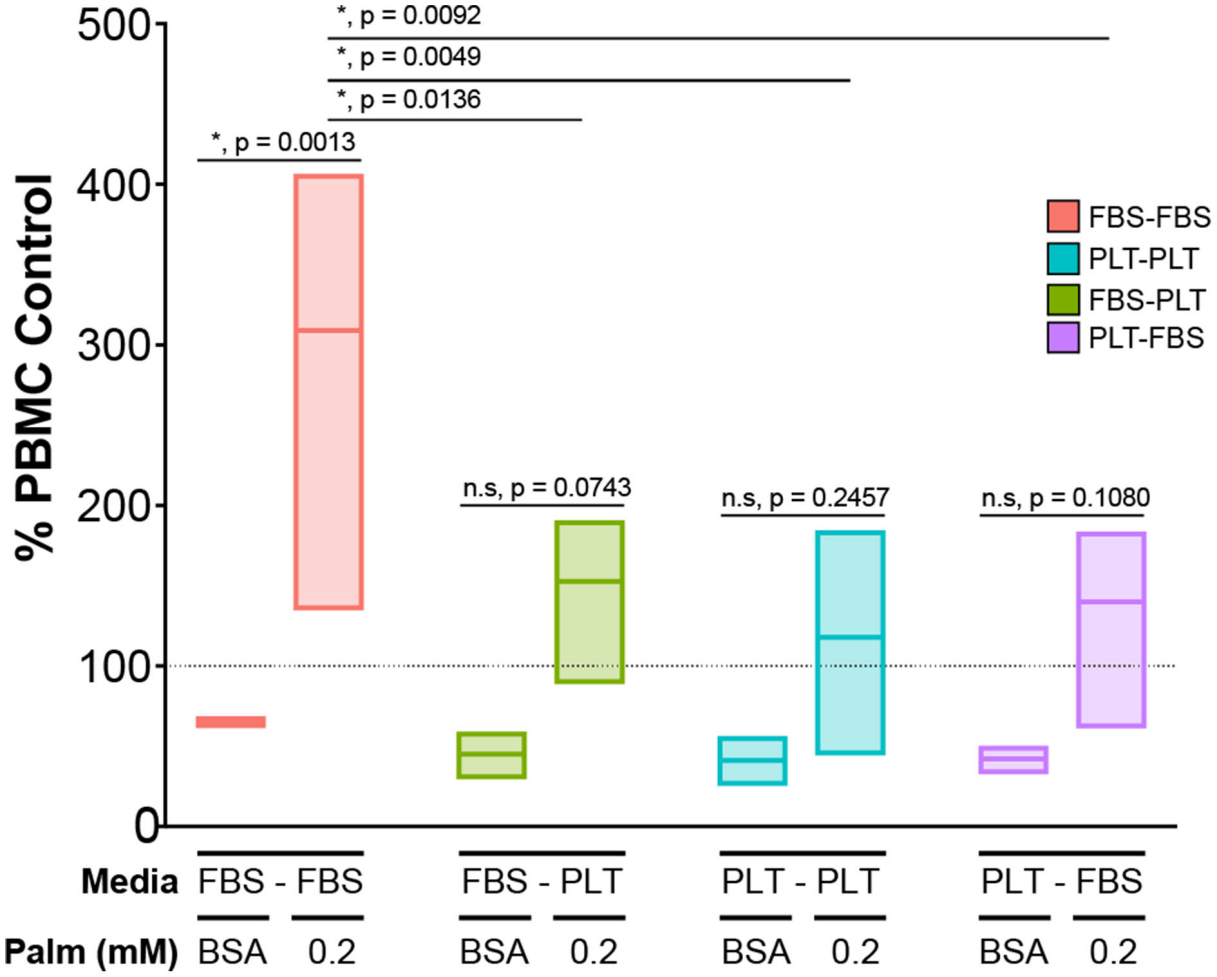
Exposure to PLT-media partially rescues ucMSCs from the palmitate-induced loss of immunosuppressive potency. CD3/CD28-stimulated CFSE-stained PBMCs were co-cultured with ucMSCs that had been maintained in (FBS-FBS, PLT-PLT) or transitioned into a new media environment (FBS-PLT, PLT-FBS) at a 1:4 ratio of ucMSC:PBMC. A single ucMSC donor (4600) was used throughout and was co-cultured with three independent PBMC donors. The dotted line represents the PBMC only control for the vehicle (BSA) and palmitate condition (0.2 mM Palm) (2-way ANOVA with Tukey correction for multiple comparisons, *n* = 3 independent PBMC donors, * denotes significance *p* < 0.05). Box-plots show the minimum, mean, and maximum values.

In line with our previous observations, FBS-FBS cells converted from an immunosuppressive phenotype in BSA to an immunostimulatory profile when exposed to palmitate (Figure 7). Interestingly, at baseline, any cell preparation that had been grown in PLT-media for a period was more immunosuppressive, on average, than FBS-FBS cells (BSA, mean ± SEM: *FBS-FBS* = 65.5 ± 1.3, *FBS-PLT* = 45.1 ± 7.7, *PLT-PLT* = 41.2 ± 8.1, *PLT-FBS* = 42.2 ± 4.3). Notably, all cell preparations grown at some stage in PLT-media were also less susceptible to the palmitate-induced immunostimulatory conversion than FBS-FBS preparations from the same donor. Although there were no statistically significant differences between the immunostimulatory levels in cell preparations grown at some point in PLT-media, PLT-PLT cells showed the lowest average level of immunostimulatory behavior compared to FBS-PLT and PLT-FBS preparations (0.2P, mean ± SEM: *FBS-PLT* = 152.7 ± 31.4, *PLT-PLT* = 117.9 ± 39.7, *PLT-FBS* = 140.0 ± 38.7). Interestingly, though PLT-FBS cells showed the most striking change in morphological features and growth kinetics in response to the transition in media supplement, these changes did not translate into a poorer performance within an immunosuppressive potency assay. The lack of synchrony between changes in morphology and changes in potency performance are particularly noteworthy given the recent interest in incorporating morphological analysis into MSC potency matrices (64, 65). PLT-media, therefore, does appear to prevent some of the damage inflicted by palmitate exposure; however, it is critical to note that growth in PLT-media does not fully restore the immunosuppressive function of ucMSCs after palmitate exposure.

## Discussion

The choice of media supplementation for expansion of MSCs can influence a range of phenotypic characteristics including growth kinetics, morphology, and multi-lineage differentiation potential (5, 9, 10, 30, 61). In industrial scale production of therapeutic MSCs, a high priority has been placed on the ability of xeno-free supplements to amplify the overall yield of MSCs, but less focus has been placed on the functional consequences of different media compositions (3, 53). In agreement with previous findings in the field (9, 10), we observed that ucMSCs grown in xeno-free conditions showed both faster growth kinetics and greater overall yields compared to their paired counterparts grown in traditional FBS-supplemented media (Figures 1B,C). An interesting early feature of ucMSC preparations grown in PLT-supplemented media was a lower overall variance in performance between donors, which is also consistent with previous findings (5). Interestingly, because cells from the same initial donor tissue were isolated and grown in two different media preparations, the decrease in donor variability suggests that variance often attributed to “inherent” donor characteristics (nature) is actually heavily influenced by early bioprocessing decisions (nurture).

Based on the clear early advantage of using a xeno-free culture system, we initially hypothesized that PLT-ucMSC preparations would show superior performance in most, if not all, of the functional assessments. However, our assessment revealed a much more nuanced effect of growing ucMSCs in PLT-media. In comparison to FBS-ucMSCs, PLT-ucMSCs showed a decrease in apoptotic induction (Figure 2), but a more dramatic drop in NAD^+^/NADH ratio (Figure 3B) in response to a metabolic stressor (palmitate exposure), while also taking up more of the fluorescent palmitate analog, BODIPY C16 (Figures 3D,E). A potential explanation for this finding is that although PLT-ucMSCs uptake higher levels of palmitate, the actual intracellular processing of palmitate might be distinct between the two preparations leading to the observed phenotypic differences. Future studies involving metabolic tracing of palmitate are needed to delineate if process-related decisions, like media supplementation, alter the intracellular handling of palmitate and contribute to the improved viability of PLT-ucMSCs after palmitate exposure. These findings are important for two reasons: first, in instances in which MSCs are persisting for long periods of time (e.g., bone graft or local injection), these findings identify the composition of the local metabolic environment as an important modifier of MSC viability and health (58), and second, that the functional response of MSCs can be modified through process-level decisions as simple as media supplementation.

Although MSCs are being explored for a range of clinical indications, the majority of current clinical trials are aiming to capitalize on the immunomodulatory axis of MSC function (68, 69). A growing number of studies have demonstrated that MSCs isolated from patients with metabolic disease have drastically altered immunomodulatory potential (32, 34, 38, 40, 47, 51); however, the potential corollary of the immunomodulatory performance of a healthy-donor MSC being altered in a “metabolically diseased” environment has not been well-established. The unfortunate underlying assumption is that the immunomodulatory potential of MSCs will be sustained, no matter the cues present within the transplant environment. However, several cues within serum, including complement (70) and TNF-α (71), have been shown to modulate MSC function, which challenges this assumption. Our recent work implicates metabolic cues, specifically physiologic levels of palmitate, as an additional and potent modifier of MSC immunomodulatory performance (52). Notably, it has also recently been reported that the reliance of MSCs on specific metabolic pathways (highly glycolytic vs. oxidative) is a critical regulator of MSC immunosuppression of T cells (72). In the present study, using a broader range of donors and a different tissue source of MSCs, we have once again found that palmitate converts MSCs from an immunosuppressive to an immunostimulatory profile (Figure 4B) when grown in standard FBS-supplemented media. Notably, we found that although xeno-free culture does not wholly prevent palmitate-induced damage, it does lessen the severity of the damage inflicted by a metabolically toxic environment. This demonstrates that bioprocess decisions have a lasting impact on the resiliency of MSC's immunomodulatory efficacy, and could be used to tailor MSCs for use within challenging metabolic environments.

The path from cell isolation to translational application of MSCs is highly variable and incorporates a number of transition points, from the choice of isolation method to the extent of time spent in culture (2). Though industrial production of MSCs has helped to standardize transition points (18, 20), the full influence of a cell's “memory” of isolation and culture conditions remains to be uncovered. In the current study, we found that ucMSCs exhibited functional memory of their original environment in some, but not all of the aspects we profiled. Growth kinetics of ucMSCs appeared to be uniquely affected by the current growth media (Figure 5B), with little effect rendered by the original media supplementation. Interestingly, regarding morphological changes, nuclear features appeared to be heavily influenced by the original growth media (Figure 6C), while cytoplasmic features were more variable in their adaptations to new environments (Figure 6B). Most important to us, any cell preparation that had ever been cultured in PLT-media, regardless of the current growth media, showed an improved suppressive profile in palmitate-rich environments (Figure 7). Therefore, a range of phenotypic changes may occur in ucMSCs in response to environmental changes, however, these phenotypic changes do not all predict functional deficits, particularly immunosuppressive potency. This finding is a notable caution for the implementation of high-throughput strategies, like image-based morphological characterization, for predicting MSC immunomodulatory performance (64, 65). The ability of morphology to predict MSC potency appears to be heavily context dependent and in our current study, the parameters we assessed proved insufficient to predict potency within palmitate-rich environments. It appears, therefore, that ucMSCs are adaptable to culture environments, but that this adaptability is not free from the “memory” of past environments. The balance achieved between “memory” and adaptation may be a result of selective expansion of highly resilient ucMSC clones isolated early in the preparation of MSCs or the result of a lasting epigenetic imprint from the early culture environment. Future studies are needed to determine by what mechanism xeno-free growth conditions protect ucMSC immunomodulatory function in challenging metabolic environments.

In conclusion, our present study has determined that both inherent donor characteristics (nature) and process-level decisions (nurture) play critical roles in the subsequent resiliency of ucMSC function in metabolically challenging environments. Although variance between donors is apparent regardless of media supplementation, xeno-free culture systems provided more consistent performance in a number of important metrics, including overall cell yield and growth kinetics. Most importantly, culturing ucMSCs in xeno-free conditions improved viability and immunosuppressive potency in response to palmitate, a common metabolic stressor present in everyone and elevated in patients with obesity and type 2 diabetes (52, 62). As MSC therapies move into clinical use for a diverse and growingly obese patient base (73), it is more critical than ever to understand the consequence of bioprocessing decisions on the subsequent performance of MSCs in complex, pathologic *in vivo* environments. By adapting *in vitro* potency models to account for disease-relevant environmental changes, critical hidden aspects of cell health and resiliency become apparent.

## Data Availability

All datasets generated for this study are included in the manuscript, supplementary files, or available upon request.

## Author Contributions

JA and LB: conceptualization and methodology. LB and JA: formal analysis. LB, AB, DB, HD, LD, JL, MS, and MF: investigation. JA and DS: resources. LB: data curation. LB, JA, AB, LD, HD, DB, JL, MS, and DS: writing and original draft. LB, JA, AB, HD, DB, LD, and MS: writing, review, and editing. LB: visualization. JA: supervision.

### Conflict of Interest Statement

While many of the reagents were provided by a Biological Industries USA Research Award, the work was conducted independently in an academic laboratory, free of the influence of any commercial or financial interest. The authors declare that the research was conducted in the absence of any commercial or financial relationships that could be construed as a potential conflict of interest.
