# Supplementary material for: Nature vs. Nurture: Defining the Effects of Mesenchymal Stromal Cell Isolation and Culture Conditions on Resiliency to Palmitate Challenge
Source: Front Immunol. 2019 May 10;10:1080. doi: 10.3389/fimmu.2019.01080 (PMC6523025; doi:10.3389/fimmu.2019.01080)
Supplement: Supplementary file 1 [file Data_Sheet_1.pdf]

Supplementary Material

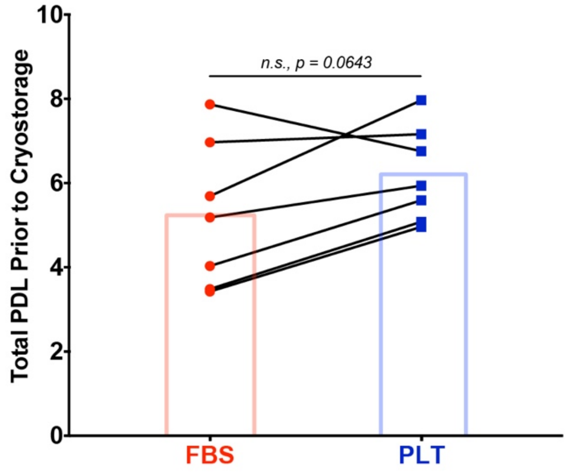

Supplemental Figure 1. Similar Total Population Doublings Were Achieved in FBS- and PLT-media Prior to Cryostorage. Total population doublings were established by hemacytometer and calculated as described in the methods section from P1-P3 outgrowths of all FBS- and PLT-ucMSC donors prior to cryostorage. Donor paired ucMSC preparations [single donor grown in either FBS-media (red circle) or PLT-media (blue square)] are represented by connected lines. Bar graph displays the mean for all donors (paired T-test, n=7 ucMSC donors, *n.s., p=0.0643*).

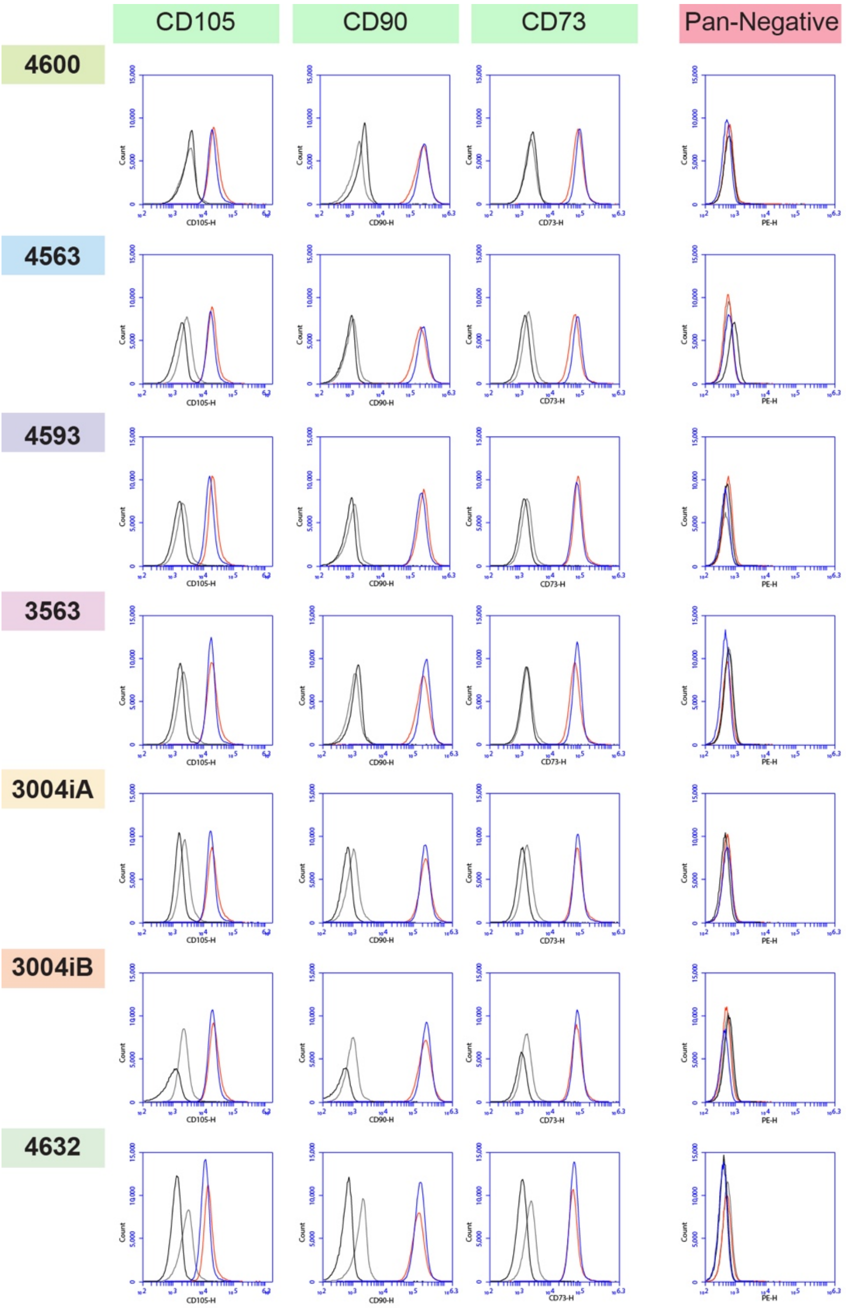

FBS Isotype
PLT Isotype
FBS
PLT

Media	CD105	CD90	CD73	Negative
FBS	98.65%	100%	100%	0.90%
PLT	99.15%	99.64%	99.67%	0.14%

Media	CD105	CD90	CD73	Negative
FBS	99.06%	99.88%	99.86%	0.41%
PLT	99.93%	99.95%	99.95%	0.02%

Media	CD105	CD90	CD73	Negative
FBS	99.81%	99.95%	99.95%	1.00%
PLT	99.92%	99.95%	99.95%	0.21%

Media	CD105	CD90	CD73	Negative
FBS	99.19%	99.98%	99.98%	0.28%
PLT	99.95%	99.97%	99.97%	0.08%

Media	CD105	CD90	CD73	Negative
FBS	99.60%	99.98%	99.98%	0.18%
PLT	99.38%	99.49%	99.49%	1.52%

Media	CD105	CD90	CD73	Negative
FBS	99.71%	99.91%	99.89%	0.29%
PLT	99.93%	99.97%	99.95%	0.07%

Media	CD105	CD90	CD73	Negative
FBS	97.54%	99.97%	99.97%	0.31%
PLT	99.92%	99.95%	99.95%	0.36%

Supplemental Figure 2. All ucMSC Preparations Meet ISCT Minimal Criteria for MSC Surface Marker Expression. Surface marker expression of CD105, CD90, CD73, CD34, CD11b, CD19, CD45, and HLA-DR was determined for all ucMSC preparations via flow cytometry. Percent positive cells were determined by setting a gate for negative markers at 2% positive for the corresponding isotype; for negative markers, a 0.5% positive gate was used.

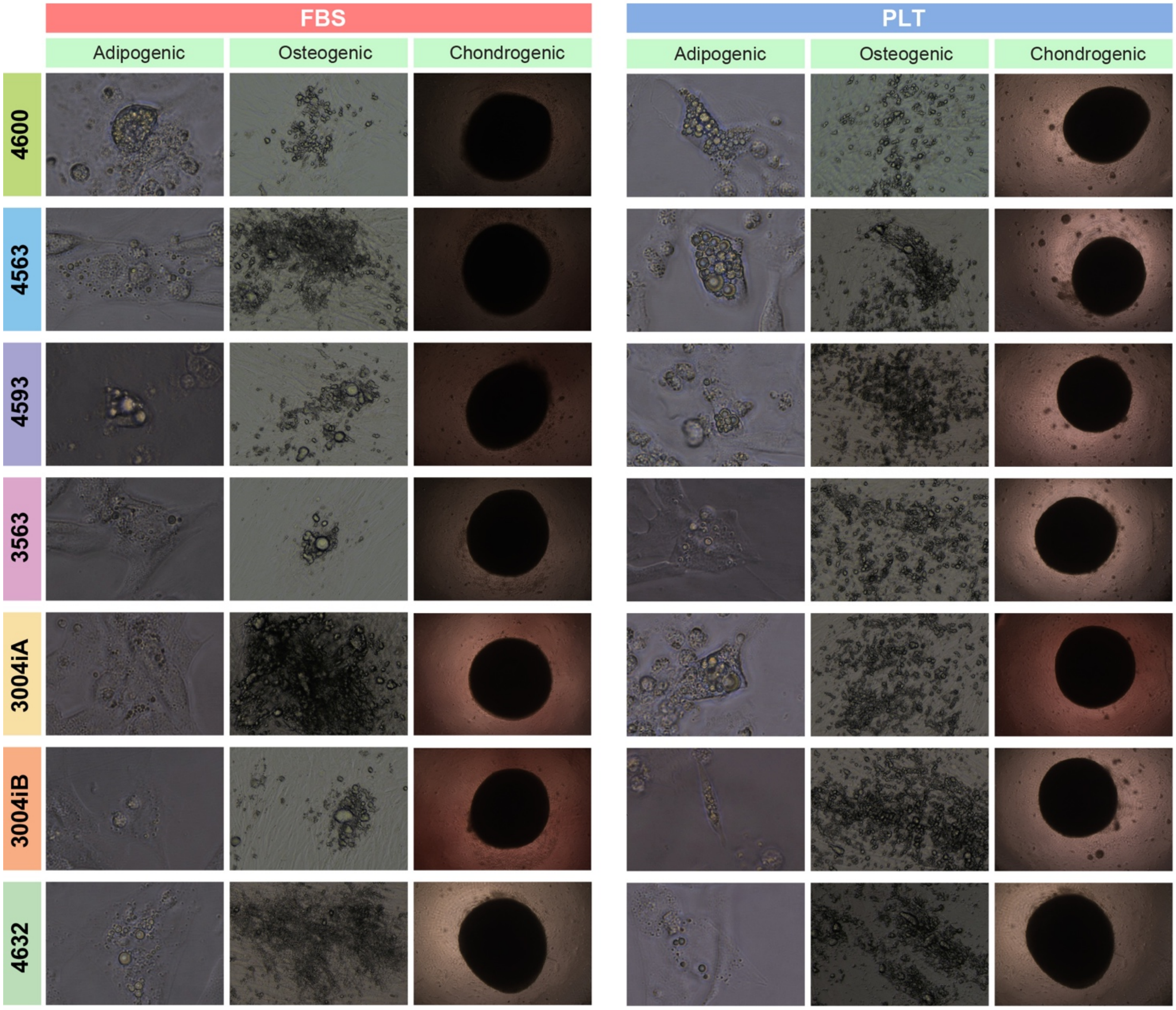

Supplemental Figure 3. FBS- and PLT-media Donors Show Features of Trilineage Potential by Brightfield Imaging. ucMSCs were imaged at 20x magnification at Day 15 of adipogenic and osteogenic differentiation and chondrogenic pellets were imaged on Day 2 of differentiation. For adipogenic differentiation, cells with lipid droplets were identified in all representative images, while for osteogenic differentiation, Alizarin Red accumulation was visible in all representative images. Chondrogenic pellets were imaged to confirm compaction of the cell pellet 48 hrs after initial plating.

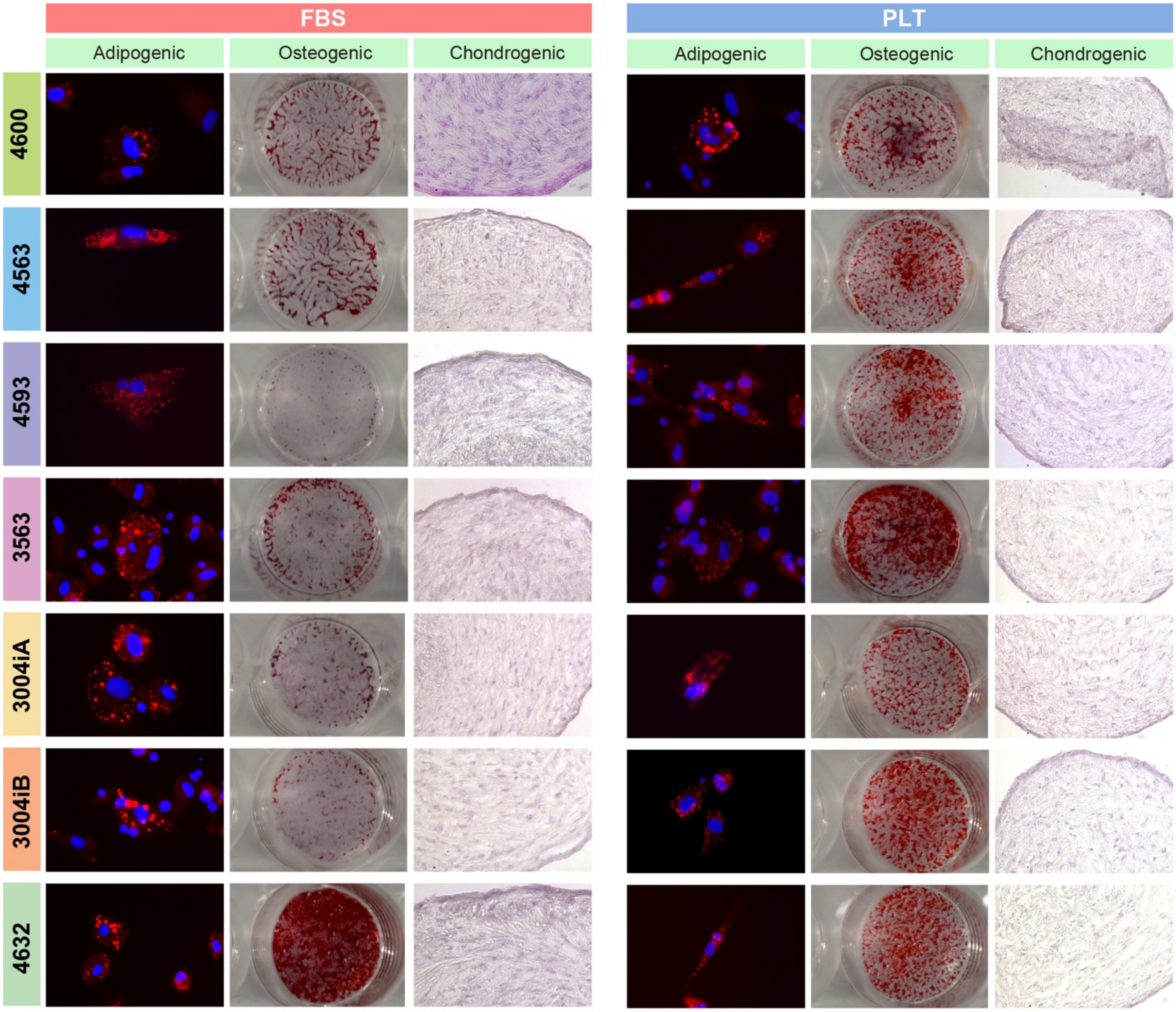

Supplemental Figure 4. UC-MSCs Show Functional Differentiation to Adipogenic, Osteogenic, and Chondrogenic Lineages Regardless of Media Supplementation. On Day 16 of differentiation, adipogenic and osteogenic cells were fixed and stained with AdipoRed (red fluorescent droplets) or Alizarin Red (red spicules), respectively. On Day 18, chondrogenic pellets were fixed, mounted with OCT, cryo-sectioned and subsequently stained with Safrinin O to confirm chondrogenic lineage.

	BSA		0.2 mM Palm-BSA		Difference	
MSC Preparation	Proliferation (%PBMC Ctl)	Granzyme B (ng/mL)	Proliferation (%PBMC Ctl)	Granzyme B (ng/mL)	Δ in Proliferation	Δ in Granzyme B (ng/mL)
4600-FBS	65.1	0.877	291.5	3.119	226.3	2.243
4600-PTL	34.2	0.377	119.1	0.257	84.8	-0.120
4563-FBS	60.5	0.338	239.4	1.017	178.9	0.679
4563-PTL	47.6	0.360	169.1	0.236	121.5	-0.125
4593-FBS	94.6	2.185	343.9	4.024	249.4	1.840
4593-PTL	77.2	1.152	317.5	3.643	240.3	2.492
3563-FBS	84.5	2.699	264.2	1.819	179.8	-0.880
3563-PTL	70.9	1.777	191.9	1.096	121.0	-0.681
3004iA-FBS	70.7	1.135	222.9	1.942	152.2	0.806
3004iA-PTL	54.7	0.708	228.8	1.427	174.0	0.719
3004iB-FBS	68.5	1.642	310.3	3.068	241.8	1.426
3004iB-PTL	33.3	0.530	93.9	0.421	60.6	-0.109
4632-FBS	58.4	1.167	344.3	2.303	285.9	1.136
4632-PTL	97.1	4.023	364.2	6.410	267.1	2.386

Supplemental Table 1. Fig. 4 data indexed by Donor ID. PBMCs, stimulated with CD3/CD28 and stained with CFSE, were exposed to fatty acid free BSA or 0.2 mM Palm-BSA in the absence (PBMC control) or presence of each MSC preparation (1:4 ratio of MSC:PBMC). After 6 days of co-culture, media was collected for granzyme B quantification and proliferation was assessed by flow cytometry. Table shows for each MSC preparation, the relative proliferation of PBMCs compared to PBMC control and the level of granzyme B detected in the culture media at the end of the co-culture. For each MSC preparation, the change in PBMC proliferation and granzyme B levels is also reported (0.2 mM Palm-BSA condition – BSA condition). Plots of data can be found in Fig. 4A and Fig. 4C.

	Media Comparison					
	FBS-FBS v. FBS-PLT	FBS-FBS v. PLT-PLT	FBS-FBS v. PLT-FBS	FBS-PLT v. PLT-PLT	FBS-PLT v. PLT-FBS	PLT-PLT v. PLT-FBS
**Cytoplasmic Feature**						
Area	n.s., p = 0.7784	n.s., p = >0.9999	****, p = <0.0001	n.s., 0.8468	****, p = <0.0001	****, p = <0.0001
Perimeter	n.s., p = 0.3069	n.s., p = 0.9998	****, p = <0.0001	n.s., p = 0.4299	****, p = <0.0001	***, p = 0.0001
Max Feret Diameter	n.s., p = 0.0781	n.s., p = 0.7059	*, p = 0.0392	n.s., p = 0.6396	****, p = <0.0001	**, p = 0.0034
Min Feret Diameter	n.s., p = 0.1088	n.s., p = 0.7602	***, p = <0.0008	n.s., p = 0.6653	****, p = <0.0001	****, p = <0.0001
Extent	**, p = 0.0019	n.s., p = 0.3095	n.s. , p = 0.204	n.s., p = 0.3421	n.s., p = 0.5102	n.s., p = 0.9947
Form Factor	*, p = 0.0465	n.s., p = 0.8424	n.s., p = 0.1789	**, p = 0.0083	***, p = 0.0001	n.s., p = 0.6708
**Nuclear Feature**						
Area	n.s., p = 0.7073	n.s., p = 0.7312	***, p = 0.0007	n.s., p = 0.2005	****, p = <0.0001	*, p = 0.0422
Perimeter	n.s., p = 0.7166	n.s., p = 0.4647	***, p = 0.0006	n.s., p = 0.0852	****, p = <0.0001	n.s., p = 0.0945
Max Feret Diameter	n.s., p = 0.9435	**, p = 0.0055	n.s., p = 0.281	**, p = 0.0014	n.s., p = 0.1192	n.s., p = 0.5281
Min Feret Diameter	n.s., p = 0.6013	**, p = 0.0011	n.s., p = 0.9755	****, p = <0.0001	n.s., p = 0.4257	*, p = 0.0139

Supplemental Table 2. 1-way ANOVA Significance Table for Differences in Morphological Features Between Media Exchange Groups. n=150-200 cells/media composition.
